# The Podocyte Power-Plant Disaster and Its Contribution to Glomerulopathy

**DOI:** 10.3389/fendo.2014.00209

**Published:** 2014-12-15

**Authors:** Janina Müller-Deile, Mario Schiffer

**Affiliations:** ^1^Division of Nephrology and Hypertension, Department of Medicine, Hannover Medical School, Hannover, Germany

**Keywords:** mitochondria, podocyte, mitochondriopathy, energy supply, diabetic nephropathy, FSGS, Nox

## Abstract

Proper podocyte function within the glomerulus demands a high and continuous energy supply that is mainly derived from the respiratory chain of the inner mitochondrial membrane. Dysregulations in the metabolic homeostasis of podocytes may result in podocyte damage and glomerular disease. This article highlights the current knowledge about podocyte energy supply by the respiratory chain. We review the regulation of mitochondrial oxidative metabolism with regard to podocytopathy and discuss the latest understanding of different mitochondrial dysfunctions of the podocyte in diabetic nephropathy and focal segmental glomerulosclerosis (FSGS). We discuss genetic forms of mitochondriopathy of the podocyte and end with recent knowledge about crosstalk between NADH and NADPH and potential therapeutic options for podocyte mitochondriopathy. We aim to raise awareness for the complex and interesting mechanisms of podocyte damage by impaired energy supply that, despite of novel findings in recent years, is poorly understood so far.

## Introduction

**Figure 1:**
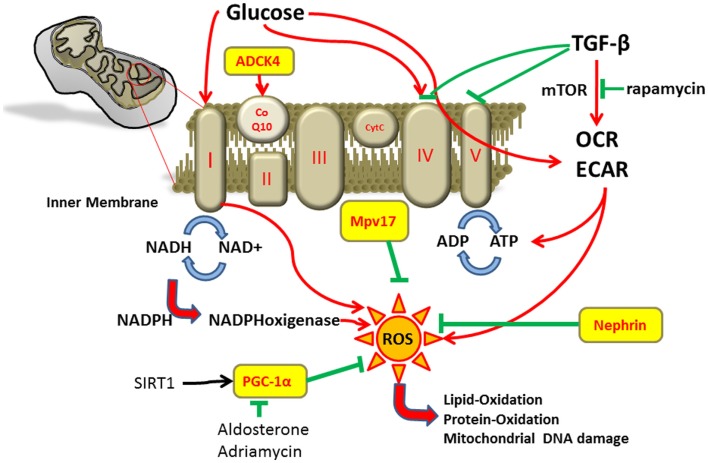
**Described pathways affecting energy metabolism and ROS production in podocytes**.

Mitochondria are essential intracellular organelles that play a major role in maintaining the energy homeostasis of cells by synthesis of adenosine triphosphate (ATP) through oxidative phosphorylation. Mitochondria are also involved in cell signaling, cellular differentiation, and control of cell cycle and growth (1). The mitochondrial respiratory chain is composed of four protein complexes. NADH dehydrogenase (complex I), cytochrome *c* reductase (complex II), cytochrome c oxidase (complex III), and cytochrome *c* (complex IV) transfer electrons and protons across the inner mitochondrial membrane generating the electrochemical gradient for ATP synthesis in complex V (Figure 1). Coenzyme Q10 is important for shuttling electrons in the respiratory chain (2–4). Some oxygen molecules are not reduced into water during oxidative phosphorylation but form reactive oxygen species (ROS) that can be converted into highly reactive radicals. These radicals can lead to oxidative damage of mitochondrial DNA, peroxidation of lipids and proteins, and activation of the mitochondrial permeability transition pores (4).

The proper function of mitochondria depends on the nuclear as well as the mitochondrial genes and the maintenance of oxidation–antioxidation balance is required for an intact intracellular signaling (5, 6).

Renal impairment in mitochondrial cytopathies most frequently involve the tubular system with De Toni-Debré–Fanconi syndrome being the most prominent example (7). However, an increase in the production of ROS has been demonstrated in experimental models of glomerular diseases too (8).

One cell type of special importance for the integrity of glomerular function is the podocyte. Podocytes are highly differentiated glomerular epithelial cells with high energetic demands. Podocyte injury results in malfunction of the glomerular filtration barrier and is a crucial step in the development of many glomerular diseases. Thus, a better understanding of the energy supply and metabolic homeostasis of podocytes seems to be crucial. This article highlights the current knowledge of podocyte energy supply by the respiratory chain and the importance of mitochondria for proper podocyte function.

## Energy Supply in Podocytes

Podocytes demand a high energy supply to maintain various cellular functions, including the organization of cytoskeletal and extracellular matrix proteins (9).

In most studies mitochondrial function was characterized using Seahorse Bioscience XF24 Extracellular Flux Analyzer to monitor cell respiration (10–12). With this technique, different metabolic states of cells can be measured in culture and blockers of different complexes of the respiratory chain can be used. Basal oxygen consumption rate of podocytes was about 55 pmol/min and the basal rate of extracellular acidification was about 3 milli-pH units/min. The complex V inhibitor oligomycin reduced oxygen consumption rate to 45% of baseline and suggested that about 55% of cellular oxygen consumption was coupled to ATP synthesis. As a complex I inhibitor reduced oxygen consumption rate to 25% of the baseline rates, mitochondrial respiration seems to be accounted for 75% of the total cellular respiration in podocytes. The rest is accounted for by proton leak (13). Proposed functions of proton leak include heat production and prevention of oxidative stress caused by ROS (14).

Similar to the aforementioned studies, we recently measured podocyte metabolic profiles under diabetic conditions in cell culture. Podocytes showed a significant increase in oxygen consumption rate after prolonged exposure to high glucose at baseline and also after treatment with the ATP synthase inhibitor oligomycin. In contrary, mitochondrial respiration was not changed in high-glucose condition in podocytes after the inhibition of respiration by rotenone (12). Measured by NADH turn over, metabolic activity of podocytes increased under TGF-β and high glucose. The observed changes in mitochondrial function may lead to metabolic conditions that sensitize the podocyte toward apoptosis. As both inhibition of glycolysis and of oxidative phosphorylation reduced ATP levels in podocytes, it was concluded that podocytes have limited ability to increase oxidative phosphorylation or glycolysis, which makes them very susceptible for dysfunctions in energy supply (12).

Furthermore, podocytes cannot increase glycolysis when mitochondrial function is blocked and thereby are not able to make up the energy deficit (13). It has to be kept in mind that this data comes from studies in isolated cultured podocytes. Conditions in the kidney where podocytes are surrounded by other glomerular cells might be different. Thus, the results from *in vitro* culture of single cell types must be interpreted with caution.

The importance of mitochondrial function in the crosstalk between different glomerular cells has been investigated by Daehn et al. (15). Their studies suggested that segmental glomerulosclerosis develops due to podocyte endothelin-β1 that acts on glomerular endothelial mitochondria and caused oxidative damage (15). Thus, the use of endothelin antagonists might be a therapeutic opportunity to rescue this podocyte–endothelial crosstalk.

## Regulation of Mitochondrial Oxidative Metabolism in Podocytes

Peroxisome proliferator-activated receptor gamma coactivator 1-alpha (PGC-1α) is a major regulator of mitochondrial oxidative metabolism (16). Silent mating type information regulation 2 homolog 1 (SIRT1) regulates PGC-1α activity and energy metabolism. Overexpression of PGC-1α or peroxisome proliferator-activated receptor-γ (PPARγ) itself protected podocyte against damage by prevention of mitochondrial dysfunction (17).

Furthermore, the PGC-1α regulated mitochondrial function is involved in doxorubicin induced podocyte injury. Doxorubicin (trade name Adriamycin) is an anthracycline antibiotic used as a drug in cancer. Its anticancer effects are believed to occur through the inhibition of topoisomerase enzyme and subsequent blockage of DNA resealing during cell replication (18). Moreover, doxorubicin reduces the expression of PGC-1α and induces mitochondrial dysfunction with increase in mitochondrial ROS production, decrease in mitochondrial DNA copy number, and reduction of ATP. On the other hand, PGC-1α overexpression was able to attenuate mitochondrial dysfunction and doxorubicin induced apoptosis of podocytes (19). The mitochondrial SIRT1–PGC-1α axis also seems to play a role in aldosterone-induced podocyte damage. Activation of this axis was able to ameliorate podocyte injury due to aldosterone (16). These findings were confirmed by further studies that showed an aldosterone dose-dependent increase in the production of ROS and reduction in nephrin expression (20). Thus, aldosterone-induced podocyte injury also seems to be mediated by mitochondrial dysfunction.

Podocyte nephrin and podocin expression was also significantly decreased in rats treated with advanced oxidation protein products leading to proteinuria (21).

Similar results come from studies with immortalized mouse podocytes where sustained application of NMDA triggered ROS production that was also associated with reduced expression of nephrin and podocin (22).

The inner mitochondrial membrane protein Mpv17 plays an important role in peroxisomal metabolism of ROS (23). Mpv17 knockout mice developed progressive glomerulosclerosis and proteinuria and died from renal failure after 9–12 months. The glomerular lesions in these mice were caused by excess of oxygen radicals and accumulation of lipid peroxidation adducts in the glomeruli (24).

In line with these findings, Mpv17 was shown to protect podocytes against oxidative stress-induced injury (25). Thus, Mpv17 seems to be important for mitochondrial homeostasis in podocytes.

## Regulation of Podocyte Mitochondria Oxidative Stress in Different Glomerular Diseases

TGF-β is an unspecific model for cell stress. After stimulation with TGF-β for 48 h, ROS levels increase by 32%. Furthermore, TGF-β also activates the mammalian target of rapamycin (mTOR) pathway and an mTOR inhibitor was able to ameliorate the TGF-β stimulated increase in oxygen consumption rate, extracellular acidification rate, and ATP (11). In our studies in cultured mouse podocytes, we could find decreased activity of cytochrome c oxidase (complex IV) and ATPase (complex V) of the respiratory chain in podocytes after treatment with TGF-β. As for glucose, we found increased NADH turnover as indicator for higher metabolic activity in podocytes in the presence of TGF-β (12).

Podocyte-specific activation of TGF-β is among others associated with endothelin-1 release. This podocyte endothelin-1 mediates mitochondrial oxidative stress and dysfunction in neighboring endothelial cells via paracrine endothelin-1 receptor type A activation. In turn, endothelial dysfunction promotes podocyte apoptosis and podocyte loss leading to albuminuria, glomerulosclerosis, and renal failure (15). Furthermore, exogenous TGF-β induces podocyte apoptosis by caspase-3 activation and is associated with upregulation of NADPH oxidase 4 (Nox 4) and increased ROS levels in mice (26).

In podocytes, Nox4 is predominantly localized to mitochondria and its upregulation markedly depolarizes the mitochondrial membrane potential. Moreover, silencing of Smad3 and Nox4 prevented mitochondrial DNA reduction, restored mitochondrial function, and decreased cellular apoptosis in puromycin aminonucleoside (PAN) treated podocytes. Thus, the Smad3–Nox4 axis might mediate mitochondrial dysfunction in podocyte damage likely via ROS generation (27).

In the following section, we would like to give examples for mitochondrial dysfunction in glomerular disease.

### Diabetic nephropathy

The most common cause for end-stage renal disease in developed countries is diabetic nephropathy, and podocyte damage is an early finding in the glomerular part of the disease. Several lines of evidence suggest that mitochondrial dysfunction plays a critical role in diabetic nephropathy.

We could show elevated activity of complex I and complex IV of the respiratory chain as well as decreased activation of complex V under acute high-glucose conditions in podocytes in previous experiments. In contrast to that, podocyte activity of complex II and III of the mitochondrial respiration chain was not affected by high glucose (12).

Mitochondria are able to change their shape, number, and intracellular distribution in response to different stimuli.

Mitochondria length and the degree to which they form closed networks are determined by the balance between fission and fusion rates of the mitochondria. Furthermore, fission and fusion are important for growth and mitochondrial redistribution (28).

Dysregulations in mitochondrial dynamics can contribute to mitochondrial dysfunction in diabetic conditions and mitochondrial fission was reported as a critical mediator of increased ROS production in hyperglycemic conditions (17, 29).

The role of Rho-associated coiled coil-containing protein kinase (ROCK) on mitochondrial dynamics was investigated by Wang et al. (30). They found that ROCK mediates hyperglycemia-induced mitochondrial fission by promoting dynamin-related protein-1 recruitment to the mitochondria (30).

Rho-associated coiled coil-containing protein kinase has a major role in mediating cytoskeleton rearrangements downstream of Rho and is important for stress fiber formation, ROS production, and cellular apoptosis (31–34). Inhibition of ROCK by fasudil ameliorated albuminuria and progression of diabetic nephropathy in a mouse model of diabetes (35).

Another example is that coagulation protease activated protein C prevented as well as accumulation of oxidative stress markers in the glomerulum as redox-regulating protein p66 in podocytes in experimental diabetic nephropathy (36). These findings further support the importance of podocyte mitochondrial homeostasis for glomerular functions.

### Nephrotic syndrome

Mitochondrial dysfunction and altered mitochondrial gene expression have also been reported in patients with nephrotic syndrome. Data supporting a critical role for mitochondria in maintaining the glomerular permeability barrier also come from studies in congenital nephrotic syndrome of the Finnish type. In this podocyte disease, mitochondria-encoded respiratory-chain components were downregulated whereas nuclear encoded subunits were unaffected. These results were interpreted as mitochondrial dysfunction with consequent abnormal production of ROS (37). Changes in nephrin expression in congenital nephrotic syndrome of the Finnish type are believed to lead to defects in the mitochondrial respiratory-chain functions resulting in excessive ROS generation and accumulation of lipid peroxidation products with consequent dysfunction of the glomerular filtration barrier (38).

Other mutations in mitochondrial respiratory-chain proteins are those of aarF domain containing kinase (ADCK) (39). ADCK have been shown to participate in coenzyme Q 10 biosynthesis. Knockdown of ADCK4 in podocytes reduced their migration phenotype. The knockdown of ADCK4 recapitulated the nephrotic phenotype in zebrafish. Moreover, a patient with steroid-resistant nephritic syndrome with a homozygous ADCK4 mutation reached partial remission under coenzyme Q10 treatment (40).

A role for podocyte mitochondrial injury in another glomerular disease was suggested by the finding that mutations in enzymes related to coenzyme Q generation and in the mitochondrial tRNA^Leu(UUR)^ are associated with focal segmental glomerulosclerosis (FSGS) (41, 42). Different gene mutations have been described to cause FSGS. Some of these mutations are related to mitochondrial function. One example is mutations in Inverted formin 2 (*INF2*) that cause monogenetic familial FSGS (43, 44). INF2 is localized in the endoplasmic reticulum and is required for efficient mitochondrial fission in mammalian cells (45). Moreover, INF2 is an actin-binding protein and is highly expressed in the podocytes where it regulates polymerization and depolymerization of the actin cytoskeleton (46). If this mitochondrial–cytoskeletal interaction is also relevant in podocytes and causes FSGS is unknown but from the discussion above it is highly speculative.

Another mitochondrial gene that was described in FSGS is prenyl diphosphate synthase subunit 2 (PDSS2). It is required for synthesis coenzyme Q10 in humans and mutations in PDSS2 have been associated with a significantly increased risk for FSGS and collapsing glomerulopathy (47). Homozygous mice carrying kidney disease mutations in the gene encoding PDSS2 (Pdss2kd/kd) develop interstitial nephritis and die from end-stage renal disease. In contrast, dietary supplementation with coenzyme Q10 rescued proteinuria and interstitial nephritis in the Pdss2kd/kd mutant mice (48).

Administration of PAN to rats produces severe proteinuria and glomerular changes, mimicking minimal changes seen in humans. Reduced mitochondrial DNA copy number led to lower levels of respiratory-chain complex I in glomeruli in PAN model (49). Furthermore, mice carrying mutant mitochondrial DNA developed FSGS and died within 6 months due to renal failure (50). In patients, A-to-G transition at mitochondrial DNA position 3243 was associated with proteinuria and FSGS (51, 52). The FSGS phenotype as the renal manifestation of mitochondrial cytopathies can even precede other manifestations of this genetic disease by many years (53, 54).

Inherited coenzyme Q2 mutation is another category of mitochondrial cytopathies, characterized by proliferation of dysmorphic mitochondria, and primary glomerular damage. This mitochondriopathy was also referred to as coenzyme Q2 nephropathy and electron microscopy showed dysmorphic mitochondria in the cytoplasm of podocytes in this disease (55).

## Potential Therapeutic Options for Podocyte Mitochondriopathy

The relevance of mitochondria for proper podocyte function makes it reasonable to look into therapeutic strategies targeting mitochondrial metabolism.

However, coenzyme Q10 mitochondriopathy is currently the only directly treatable mitochondrial defect (56). But recent findings in interactions of podocytes metabolism give hope for further substances to improve podocyte mitochondrial impairment.

First of all, we want to review the fundamental difference between NADH and NADPH in biochemical reactions that should not be mixed up. NADH is generated in a catabolic process from glycolysis and citrate cycle whereas NADPH is a reducing agent and donor for electrons and proteins. Mitochondria are able to generate NADPH by the transfer of reducing equivalents from NADH to NADP^+^. NADPH can be used to reduce glutathione within the mitochondrion and thereby controlling mitochondrial ROS (57).

Nox is prominently expressed in podocytes and is another source of ROS production (58). An overproduction of ROS was found in many podocyte injury models such as diabetic nephropathy, membranous glomerular nephropathy, minimal change disease, and FSGS (59).

Deficiencies of the vitamins B6, B9, and B12 can lead to high homocysteine. Hyperhomocysteinemia-induced Nox activation was shown to be important for inflammatory mechanisms through NOD-like receptor protein-dependent inflammasomes (60). Moreover, Nox subunits aggregated and were activated by lipid raft clustering and therefore were suggested to be a leading mechanism in hyperhomocysteinemia-related oxidative injury of podocytes. These injuries include reduced expression of slit diaphragm molecules, cytoskeleton disarrangement, and cell apoptosis (61, 62).

Mitochondria serve high levels of antioxidants and are able to limit Nox activity. Mitochondria target ROS produced by Nox but are also a significant source of ROS themselves. The cross talk between mitochondria and Nox may represent a vicious cycle of ROS production and it already has been tried to influence this cross talk pharmacologically in mitochondrial dysfunctions of podocytes.

Nox inhibition by diphenylene iodonium and apocynin could attenuate podocyte injury (63). The mitochondrial DNA copy number in podocytes is reduced in PAN model and consequently leads to increase in oxidative stress and shortage of ATP synthesis. Nox inhibition by apocynin decreased not only superoxide production in podocytes but also inhibited endocytosis and urinary albumin excretion in PAN rats (64). Nox is also the main source of ROS in diabetic podocytes and contributes to the development of diabetic nephropathy and metformin could decrease production of ROS through reduction of NADPH oxidase activity (64). Another example for enzymes that regulate mitochondria dysfunction and cause podocytopathy are glycogen synthase kinase (GSK) 3β. An inhibitor of GSK3β ameliorated proteinuria, attenuated podocyte injuries, foot process effacement, and glomerulosclerosis by alleviating mitochondria damages in podocytes (65). In studies, eplerenone treatment could prevent ROS overproduction in aldosterone infused rats (66).

Furthermore, statins also seem to have beneficial effects on mitochondrial function in podocytes. Rosuvastatin attenuated angiotensin II-dependent increases in Nox activity and ROS generation in cultured podocytes (67).

The Dahl salt sensitive rat is a rodent model of hypertension that has many similarities with hypertension in human. The rats develop salt sensitive hypertension, impaired renal function, and proteinuria. In these rats, pre-treatment with pitavastatin was podocyte protective by preventing NADPH subunits expression (68). These findings are of special interest under the hypothesis of an interaction of NADPH and mitochondrial energetic metabolisms. The PPAR-γ agonist rosiglitazone is an antidiabetic drug that works as an insulin sensitizer. Rosiglitazone was also able to protect podocytes against damage by prevention of mitochondrial dysfunction (22).

## Conclusion

In podocytes, proper mitochondrial function is indispensable for maintenance and function of the glomerulus. Podocyte mitochondriopathy is involved in many different acquired and genetic glomerular diseases and influencing energy supply and antagonizing ROS production might be a potential therapeutic strategy in the future.

## Conflict of Interest Statement

The authors declare that the research was conducted in the absence of any commercial or financial relationships that could be construed as a potential conflict of interest.
